# A high neutrophil-to-platelet ratio is associated with hematoma expansion in patients with spontaneous intracerebral hemorrhage: a retrospective study

**DOI:** 10.1186/s12883-023-03055-3

**Published:** 2023-01-18

**Authors:** Yujian Li, Xiang Yang, Huiqing Zhou, Xuhui Hui, Hao Li, Jun Zheng

**Affiliations:** 1 Department of Neurosurgery, West China Hospital, Sichuan University, 37 Guo Xue Xiang, Wu Hou District, 610041 Chengdu, P.R. China; 2 Department of Intensive Care Unit, Fourth People’s Hospital of Sichuan Province, Chengdu, P.R. China

**Keywords:** Hematoma expansion, Inflammation, Intracerebral hemorrhage, Neutrophil-to-platelet ratio, Predictor

## Abstract

**Background:**

Early hematoma expansion (HE) occurs in 20 to 40% of spontaneous intracerebral hemorrhage (ICH) patients and is a primary determinant of early deterioration and poor prognosis. Previous studies have shown that inflammation is a major pathological feature of ICH, and the neutrophil-to-platelet ratio (NPR) is a marker of systemic inflammation. Therefore, we aimed to assess the association between the NPR and HE in ICH patients.

**Methods:**

We retrospectively collected and analyzed data from ICH patients who received treatment at our institution from January 2018 to November 2019. The NPR was calculated from the admission blood test. Brain computed tomography (CT) scans were performed at admission and repeated within 24 h. Hematoma growth was defined as relative growth > 33% or absolute growth > 6 ml.

**Results:**

A total of 317 patients were enrolled in our study. Multivariate logistic regression analysis indicated that the NPR was an independent predictor of HE [odds ratio (OR) = 1.742; 95% CI: 1.508–2.012, *p* < 0.001]. Receiver operating characteristic (ROC) curve analysis revealed that the NPR could predict HE, with an area under the curve of 0.838 (95% CI, 0.788–0.888, *p* < 0.001). The best predictive cut-off of the NPR for HE was 5.47 (sensitivity, 75.3%; specificity, 77.6%).

**Conclusions:**

A high NPR was associated with an increased risk of HE in patients with ICH.

## Background

Spontaneous intracerebral cerebral hemorrhage (ICH) comprises approximately 10 to 20% of all strokes, with high rates of disability and mortality [1]. Early hematoma expansion (HE) occurs in 20 to 40% of ICH patients and is a primary determinant of early deterioration and poor prognosis [2]. Previous studies have shown that inflammation is a major pathological feature of ICH [3–5]. In addition, systemic inflammatory responses have been found to be associated with the pathological process of active bleeding in ICH patients [6]. The neutrophil-to-platelet ratio (NPR) is a marker of systemic inflammation. A previous study indicated that the interaction between neutrophils and platelets plays a role in vascular injury after cerebral infarction [7]. Moreover, He et al. conducted a retrospective study of 279 ischemic stroke patients with hemorrhagic transformation and found that a high NPR was an independent predictor of hemorrhagic transformation [8].

However, the literature regarding the relation between the NPR and HE in ICH patients is infrequent. Therefore, this study aimed to explore the association between the NPR at admission and early HE after spontaneous ICH.

## Methods

### Patients and definitions

We performed a retrospective review of patients with ICH who visited West China Hospital from January 2018 to November 2019. The study protocol was approved by the ethics committee of our hospital. Informed consent was obtained from all patients or family members. We defined the inclusion criteria as follows: 1) a diagnosis of intracranial hemorrhage by computed tomography (CT); 2) patient underwent the first CT scan on admission and the second CT scan at 24 h after the onset of symptoms; 3) routine examinations and laboratory blood tests were conducted within 24 h after admission; and 4) patient age ≥ 18 years. We excluded patients with 1) ICH attributable to aneurysm, arteriovenous malformation or moyamoya disease; 2) ICH attributable to acute cerebral infarction, thrombolysis of cerebral or myocardial infarction; 3) prior systemic diseases such as cancer, hematological diseases, immunological disease, neurological disease, recent infectious disease, severe hepatic dysfunction, renal dysfunction and coagulation dysfunction; 4) a medical history of anticoagulant use or antiplatelet treatments; 5) patients who underwent surgery before the 24-h CT; and 6) isolated intraventricular hemorrhage.

### Clinical manifestation assessment

Baseline clinical and demographic parameters were collected at hospital arrival, including age; sex; Glasgow Coma Scale (GCS) score on admission; National Institutes of Health Stroke Scale (NIHSS) score on admission; blood pressure; cigarette consumption and alcohol use; and the medical history, including that of ischemic stroke, ICH, hypertension and diabetes mellitus.

### Laboratory examinations

Radiological data were recorded, including hematoma location, hematoma size and the presence of intraventricular hemorrhage (IVH). Laboratory variables were also recorded, including red blood cell (RBC) count, hemoglobin, absolute neutrophil count (ANC), absolute lymphocyte count (ALC), absolute monocyte count (AMC), platelet count, prothrombin time (PT), activated partial thromboplastin time (APTT), international normalized ratio (INR) and blood glucose level. Admission NPR was calculated as the ratio of the ANC × 100 to the platelet count.

### Outcome assessments

Two reviewers independently evaluated all the head CT scans. Any disagreement between the two reviewers was resolved by consensus. All patients underwent the first CT scan at admission, and the follow-up CT scan was performed within 24 h of symptom onset. Hematoma volume was measured by the ABC/2 method as described previously [9]. HE was defined as absolute growth of > 6 ml or relative growth of > 33% from the first CT to the follow-up CT [10].

### Statistical analysis

The clinical data, laboratory parameters and imaging characteristics of ICH patients with and without HE were compared. Continuous variables are expressed as mean ± standard deviation or median with interquartile range (IQR) for normally distributed and non-normally distributed variables, respectively, whereas categorical variables are expressed as frequency and percentage. Univariate analyses were conducted by independent t-test, Mann–Whitney U-test, chi-square (χ^2^) test, or Fisher’s exact test. Independent t-tests or Mann–Whitney U-tests were applied to compare continuous variables. The chi-square (χ^2^) test or Fisher’s exact test was used to compare categorical data. Variables with a *p*-value < 0.10 on univariate analysis were entered into a multivariate regression model. To facilitate interpretation, some variables were classified as follows: GCS score as “13–15 points”, “9–12 points” and “3–8 points” and hematoma location as “infratentorial hematoma” and “supratentorial hematoma”. Receiver operating characteristic (ROC) curve analysis was performed to indicate the value of the NPR for the prediction of HE in ICH patients. The cut-off value of the NPR was set by the Youden index from the ROC curve. A value of *p* < 0.05 was considered to be significant. All the above mentioned statistical analyses were carried out with SPSS version 21.0 (SPSS, Chicago, IL, USA).

## Results

**Fig. 1:**
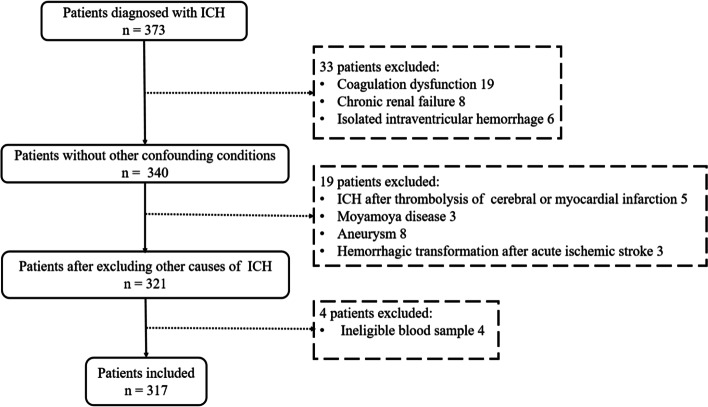
Flowchart of study enrollment

A total of 317 consecutive ICH patients (230 males and 87 females) meeting the inclusion criteria were enrolled in this study (Fig. 1). The median age, median baseline hematoma volume and median NPR were 56 years (IQR, 45–67.5), 24.00 ml (IQR, 10.15–41.84) and 4.79 (IQR, 2.70–6.63), respectively. HE was detected in 89 (28%) patients. For these patients, the median age was 58 (IQR, 49–70) years, 61 (68.5%) were men and 28 (31.5%) were women. The median NPR of the HE group was significantly higher than that of the group without HE (*p* < 0.001) (Table 1).

Univariate analysis showed significant associations between HE with age (*p* = 0.028), GCS score on admission (*p* < 0.001), NIHSS score on admission (*p* = 0.019), baseline hematoma volume (*p* < 0.001), RBC count (*p* = 0.03), ANC (*p* < 0.001), ALC (*p* < 0.001), AMC (*p* = 0.003), Platelet (*p* < 0.001), NPR (*p* < 0.001), PT (*p* = 0.009), INR (*p* = 0.005) and blood glucose (*p* < 0.001) (Table 1).

The multivariate analysis indicated that GCS score (3–8 points) [OR = 3.387, 95% CI: 1.443–7.949, *p* = 0.005], NPR (OR = 1.742; 95% CI: 1.508–2.012, *p* < 0.001) and larger hematoma size (OR = 1.015, 95% CI: 1.002–1.028, *p* = 0.022) were significantly correlated with HE (Table 2).

**Fig. 2:**
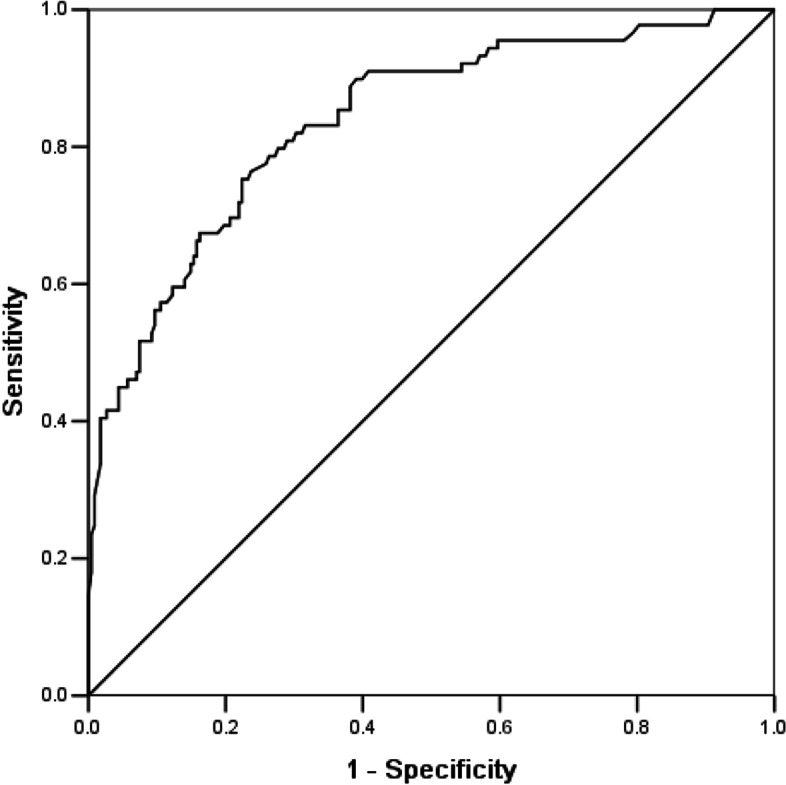
Receiver operating characteristic curve for the value of the NPR for the prediction of HE in ICH patients

The area under the ROC curve was 0.838 (95% CI, 0.788–0.888, *p* < 0.001) for HE (Fig. 2). The best predictive cut-off value of HE was 5.47 (sensitivity, 75.3%; specificity, 77.6%).

## Discussion

This study, to the best of our knowledge, is the first to analyze the relationships between the NPR and HE in spontaneous ICH patients. The present study found that a high NPR was related to HE after spontaneous ICH.

The occurrence of HE could be detected within 3 h of symptom onset in approximately 73% of ICH patients, and clinically obvious expansion was present in 35% of patients [11–13]. Accumulating evidence suggests that inflammation caused by HE accelerates brain injury in patients with ICH [4, 14–16]. Although the relationship between WBC count and outcome in ICH patients has been well demonstrated [3, 17], the correlation between early HE and leukocyte subsets remains disputed [4, 16, 18, 19]. In our study, the univariate analysis showed significant associations between HE and ANC, ALC, AMC and NPR, and multivariate logistic regression analysis showed that all those biomarkers mentioned above could not independently predict early HE, except for the NPR. However, the exact underlying mechanism of the associations between routine blood variables and HE remains unclear and needs further study.

Neutrophils play a basic defensive role in both infection-related diseases and aseptic inflammation, which are indicators of inflammation and immune response [20]. Neutrophils appear first in the hematoma [21], delivering pro-inflammatory factors, oxygen free radicals and proteases, which could have an effect on blood–brain barrier (BBB) disruption and brain damage [22, 23]. Previous studies have reported that neutrophils are the primary cellular source of metalloproteinases (MMPs), specifically MMP-9, working on the BBB [24]. Moreover, it has been shown that in ischemia–reperfusion injury, the increase in BBB permeability induced by WBC-derived MMP-9 is associated with peak neutrophil infiltration [25]. Furthermore, accumulating studies have reported that HE is associated with changes in the basal membrane of the BBB induced by MMP [4, 26, 27]. Therefore, it is reasonable to believe that neutrophils are associated with HE.

Several studies have reported the relationship between neutrophil count and hematoma size. Neutrophil count at admission was positively associated with intracerebral hemorrhage volume [28], and it was found in another study that the inhibition of neutrophil recruitment could reduce the amount of bleeding [29]. One study reported that neutrophil count was negatively related to an increased risk of HE during the hyperacute phase of ICH [19]. One possible explanation for this paradoxical finding is that the injury to blood vessels caused by neutrophils may be mediated by platelets, and neutrophil-platelet interactions may play different roles in vascular inflammation at different stages of ICH [30]. Despite activated neutrophils having a procoagulant role [31], the mutual relationship between neutrophils and platelets could increase the formation of reactive oxygen species and aggravate vascular damage [32]. In addition, platelets, as a considerable contributor to some pro-inflammatory factors, could enhance the aggregation of activated neutrophils [30, 33].

In a review of neutrophil-platelet interplay, activated platelets were found to be connected with the release of inflammatory mediators, the accumulation of neutrophils, and increased vascular permeability [7]. By locally releasing soluble vascular protective factors, platelet-endothelial interplay may prevent or treat neutrophil-induced vascular damage [34]. The hemostatic role of platelets depends on embolization and coagulation at the location of vascular injury [35], which contributes to the preservation the integrity of the BBB [36]. Systemic inflammation is often accompanied by thrombocytopenia, which may be attributed to the immune response in the blood circulation [30]. According to the above, it can be speculated that in patients with ICH, the higher the NPR, the more serious the BBB damage may be, resulting in the higher occurrence of HE.

The NPR may be more stable as a ratio than individual blood parameters, such as neutrophils or thrombocytes, because of the mutual relationship between neutrophils and platelets. Recent studies have shown a correlation between the NPR and other diseases, such as ischemic stroke [8, 37–40], ST-elevation myocardial infarction [41] and infective endocarditis [42]. For example, a high NPR was related to an increased risk of hemorrhagic transformation in acute ischemic stroke patients [8]; the platelet-to-neutrophil ratio (PNR) was found to be an independent protective predictor of 90-day prognosis in patients with acute ischemic stroke [38]. Moreover, the PNR on admission could independently predict poor functional prognosis in ischemic stroke patients undergoing intravenous thrombolysis [39]. Consistent with these findings, we found a significant association between the NPR and ICH, and a high NPR on admission was an independent predictor of HE in ICH patients.

In addition, previous studies have reported that a larger baseline ICH volume and lower GCS score on admission were associated with HE [43, 44]. In our study, the initial hematoma volume and GCS score on admission were predictors of HE in both univariate analysis and multivariate regression analysis. The results were consistent with Zhang’s study [44] and Li’s study [45]. On the other hand, an onset-to-first-CT time of less than 6 h was independently associated with HE [19, 46]. However, in our study, the median onset-to-first-CT time was 7 h and the median for the group with HE was 8 h. One possible explanation may be that West China Hospital of Sichuan University is an upper-level hospital in Southwest China, and many patients request to be transferred to our hospital for treatment from lower-level hospitals in this region or other regions. Therefore, the median onset-to-first-CT time in our study was longer.

Given HE being the independent risk factor for disability and death in ICH patients, it is crucial to timely identify HE [2, 13]. In this case, NPR has its clinical implications. Firstly, in clinical practice, as a routine indicator of blood test, it is easy and convenient to obtain NPR. Moreover, the predictability of NPR here can help clinicians initially estimate the risk of HE in ICH patients, and then conduct appropriate treatment and follow-up CT for that population. In addition, since single predictor for HE has its limitation, there are currently a variety of prediction scores containing several predictors [47]. Similarly, the combination of NPR and other predictive factors can form new prediction scores with higher specificity and sensitivity. However, the potential clinical implications of NPR should be further investigated.

Several limitations should be noted in this study. First, the data were recorded in a single center, and the sample size was limited. Second, although the results of this retrospective study may be influenced by confounding factors, multivariate analysis was used to address this problem. Third, given the complex role of inflammation in HE, more studies that record more inflammatory biomarkers are necessary in future studies.

## Conclusions

This present study showed that a high NPR was related to the risk of HE in ICH patients. These findings may assist clinicians in identifying ICH patients who have increased risk for HE and then in conducting appropriate treatment and follow-up CT for that population. In view of the limitations of the study, future well-designed studies are needed to confirm our findings.

**Table 1:** Univariate analysis of clinical characteristics related to HE in patients with ICH

Characteristic	Total (*n* = 317)	HE (*n* =89)	No HE (*n* =228)	*p*-Value
Age(years)	56(45,67.50)	58(49,70)	54(43,66)	0.028*
Sex (male)	230(72.6%)	61(68.5%)	169(74.1%)	0.329
Hypertension	258(81.4%)	76(85.4%)	182(79.8%)	0.335
Diabetes mellitus	26(8.2%)	6(6.7%)	20(8.8%)	0.653
Prior ischemic stroke	10(3.2%)	2(2.2%)	8(3.5%)	0.826
Prior ICH	11(3.5%)	4(4.5%)	7(3.1%)	0.779
Smoking	98(30.9%)	23(25.8%)	75(32.9%)	0.279
Alcohol consumption	83(26.2%)	19(21.3%)	64(28.1%)	0.256
Onset-to-first-CT time(hours)	7(4,15)	8(5,14.5)	7(4,15)	0.249
SBP(mmHg)	163 ± 27	162 ± 26	164 ± 27	0.664
DBP(mmHg)	96 ± 18	96 ± 16	97 ± 19	0.578
GCS score on admission	-	-	-	< 0.001**
13-15	114(36.0%)	15(16.9%)	99(43.4%)	-
9-12	104(32.8%)	29(32.6%)	75(32.9%)	-
3-8	99(31.2%)	45(50.6%)	54(23.7%)	-
NIHSS on admission	12(9,14)	12(10,14)	10(9,13)	0.019*
Infratentorial hematoma	67(21.1%)	20(22.5%)	47(20.6%)	0.760
Hematoma size (ml)	24.00(10.15,41.84)	35.24(14.98,52.20)	21.23(8.36,38.18)	<0.001**
Presence of IVH	133(42.0%)	42(47.2%)	91(39.9%)	0.256
RBC, x10^12^	4.58 ± 0.72	4.44 ± 0.75	4.63 ± 0.70	0.031*
Hemoglobin(g/L)	139 ± 20	135 ± 21	140 ± 19	0.061
Neutrophil, x10^9^	8.12(5.25,11.33)	10.86(8.56,13.74)	6.85(4.74,9.93)	<0.001**
Lymphocyte, x10^9^	1.22(0.79,1.83)	0.91(0.65,1.29)	1.31(0.87,1.93)	<0.001**
Monocyte, x10^9^	0.51(0.38,0.73)	0.63(0.40,0.81)	0.50(0.35,0.69)	0.003*
Platelet, x10^9^	185 ± 64	159 ± 60	195 ± 62	<0.001**
NPR	4.79(2.70,6.63)	7.37(5.46,9.67)	3.85(2.50,5.40)	<0.001**
PT (s)	10.9(10.5,11.4)	11.1(10.7,11.5)	10.9(10.4,11.3)	0.009*
APTT (s)	26.1(24.4,27.6)	25.4(24.2,27.6)	26.3(24.6,27.6)	0.224
INR	0.93(0.89,0.97)	0.95(0.91,1.00)	0.92(0.89,0.97)	0.005*
Blood glucose (mmol/L)	7.85(6.37,9.69)	8.68(7.08,11.87)	7.60(6.31,9.23)	<0.001**

Values are n (%) and median (25,75%)

*HE* hematoma expansion, *ICH* intracerebral hemorrhage, *SBP* systolic blood pressure, *DBP* diastolic blood pressure, *GCS* Glasgow coma scale, *NIHSS* National Institutes of Health stroke scale, *IVH* intraventricular hemorrhage, *RBC* red blood cells, *NPR* neutrophil to platelet ratio, *PT* prothrombin time, *APTT* activated partial thromboplastin time, *INR* international normalized ratio

*p < 0.05. **p < 0.001

**Table 2:** Multivariate analysis of predictors for HE

Predictors	OR (95% CI)	*p*-Value
Age(years)	0.998 (0.975-1.021)	0.857
NPR(per 1 increase)	1.742 (1.508-2.012)	<0.001**
GCS score on admission	-	**-**
GCS (13-15 points)	Reference	**-**
GCS (9-12 points)	1.796 (0.772-4.183)	0.174
GCS (3-8 points)	3.387 (1.443-7.949)	0.005*
NIHSS on admission	1.020(0.927-1.122)	0.688
PT (s)	0.929 (0.354-2.441)	0.882
RBC, x10^12^	0.967 (0.446-2.098)	0.932
Hemoglobin(g/L)	1.003 (0.985-1.020)	0.772
Lymphocyte, x10^9^	0.727 (0.457-1.158)	0.180
Monocyte, x10^9^	2.003 (0.616-6.508)	0.248
Neutrophil, x10^9^	0.926 (0.715-1.199)	0.561
Platelet, x10^9^	1.001 (0.994-1.007)	0.842
INR	3.230 (0.024-430.0)	0.639
Blood glucose (mmol/L)	1.073 (0.981-1.174)	0.125
Hematoma size (per 1 ml increase)	1.015 (1.002-1.028)	0.022*

*OR* odds ratio, *CI* confidence interval, *HE* hematoma expansion, *NPR* neutrophil to platelet ratio, *GCS* Glasgow coma scale, *NIHSS* National Institutes of Health stroke scale, *PT* prothrombin time, *RBC* red blood cells, *INR* international normalized ratio

*P < 0.05. **P < 0.001

## Data Availability

The datasets used and/or analysed during the current study available from the corresponding author on reasonable request.

## Abbreviations

ICH: Intracerebral cerebral hemorrhage
HE: Hematoma expansion
NPR: Neutrophil-to-platelet ratio
CT: Computed tomography
GCS: Glasgow Coma Scale
NIHSS: National Institutes of Health Stroke Scale
IVH: Intraventricular hemorrage
RBC: Red blood cell
AMC: Absolute monocyte count
ANC: Absolute neutrophil count
ALC: Absolute lymphocyte count
PT: Prothrombin time
APTT: Activated partial thromboplastin time
INR: International normalized ratio
IQR: Interquartile range
ROC: Receiver operating characteristic
BBB: Blood–brain barrier
MMPs: Metalloproteinases
PNR: Platelet-to-neutrophil ratio
